# Antitumor Activity of All-Trans Retinoic Acid and Curcumin-Loaded BSA Nanoparticles Against U87 Glioblastoma Cells

**DOI:** 10.3390/life16010131

**Published:** 2026-01-15

**Authors:** Ceyda Sonmez, Aleyna Baltacioglu, Julide Coskun, Gulen Melike Demirbolat, Ozgul Gok, Aysel Ozpinar

**Affiliations:** 1 Department of Biochemistry and Molecular Biology, Graduate School of Health Sciences, Acibadem Mehmet Ali Aydinlar University, Istanbul 34752, Türkiye; ceyda.sonmez@live.acibadem.edu.tr (C.S.); aleyna.baltacioglu@live.acibadem.edu.tr (A.B.); 2 Acibadem Labmed Clinical Laboratories, Istanbul 34752, Türkiye; julide.coskun@acibademlabmed.com.tr; 3 Department of Pharmaceutical Technology, Faculty of Pharmacy, Acibadem Mehmet Ali Aydinlar University, Istanbul 34752, Türkiye; melike.demirbolat@acibadem.edu.tr; 4 Department of Biomedical Engineering, Faculty of Engineering and Natural Sciences, Acibadem Mehmet Ali Aydinlar University, Istanbul 34752, Türkiye; ozgul.gok@acibadem.edu.tr; 5 Department of Medical Biochemistry, School of Medicine, Acibadem Mehmet Ali Aydinlar University, Istanbul 34752, Türkiye

**Keywords:** glioblastoma, U87-MG, all-trans retinoic acid, curcumin, BSA nanoparticles, drug delivery, apoptosis, cell migration

## Abstract

Glioblastoma (GBM) is a highly aggressive brain tumor characterized by invasive growth, intrinsic drug resistance, and the presence of the blood–brain barrier. All of these features make treatment extremely challenging and underscore the need for developing effective combination strategies and advanced drug delivery systems. This study aimed to develop a bovine serum albumin (BSA) nanoparticle (NP)-based delivery system to overcome the poor bioavailability and pharmacokinetic limitations of two potent anti-tumor agents, all-trans retinoic acid (ATRA) and curcumin (CURC), and to evaluate their antitumor activity in U87-MG GBM cells. Drug-free and ATRA/CURC-loaded BSA-NPs were synthesized using an optimized desolvation method and characterized in terms of particle size, polydispersity index, morphology, drug encapsulation efficiency, and release behavior. The cytotoxic, anti-migratory, and pro-apoptotic effects of the NPs on U87-MG GBM cells were assessed using real-time proliferation and migration assays and Annexin V/PI staining followed by flow cytometry. Collectively, the findings indicated that the co-delivery of ATRA and CURC using BSA-NPs showed enhanced antiproliferative, antimigratory, and pro-apoptotic effects. With its controlled release profile, high loading capacity, and favorable nanoscale dimensions, the ATRA-CURC-BSA–NP system represents a promising nanoplatform for GBM therapy that warrants further in vivo investigation. To the best of our knowledge, this is the first study demonstrating the inhibition of glioblastoma cell growth through the co-delivery of all-trans retinoic acid and curcumin using a bovine serum albumin-based nanoparticle system.

## 1. Introduction

Glioblastoma (GBM), characterized by its aggressive and invasive nature, is the most common and lethal brain tumor affecting humans. The World Health Organization (WHO) classifies GBM as a grade IV tumor among all central nervous system neoplasms, with an incidence rate of ~7 per 100,000, which may increase with age [1,2]. Intratumoral heterogeneity and invasive growth potential contribute to the extremely poor prognosis of GBM, with a 5-year survival rate of <7% and an average survival time of only 12–18 months [3,4]. Despite existing standard therapies such as surgical resection, radiation, and temozolomide (TMZ)-based chemotherapy, GBM remains one of the most challenging malignancies to successfully treat, owing to its highly invasive characteristics, complex tumor microenvironment (TME), and considerable drug resistance [5]. The severe infiltration of tumor cells into the brain renders complete surgical resection practically impracticable, while the blood–brain barrier (BBB) and intratumoral molecular heterogeneity substantially restrict effective and localized medication delivery [2,6]. Given the aggressive pathology of GBM, low survival rates, poor therapeutic responses, and resistance to current treatments, the integration of combination drug strategies with advanced drug delivery systems is considered a promising approach to enhance GBM treatment efficacy.

The disruption of fundamental cell signaling pathways, including p53, RTK/Ras/PI3K, Rb/E2F, Notch, STAT3, and Wnt/β-catenin, facilitate the emergence of glioblastoma stem cells (GSCs), which, in turn, drive tumor growth, enhance invasiveness through epithelial–mesenchymal transition (EMT), enable immune evasion, and contribute to therapeutic resistance [7,8,9,10]. The pro-inflammatory and immunosuppressive TME exacerbates the aggressive characteristics of GBM, thereby restricting treatment effectiveness [11,12]. All-trans retinoic acid (ATRA) and curcumin (CURC) are powerful anti-tumor agents that influence critical aspects of GBM pathogenesis, including proliferation, invasion, inflammation, and oxidative stress, through distinct biochemical pathways [13,14,15,16,17,18,19,20,21]. ATRA facilitates differentiation, inhibits EMT, and restructures the TME. These effects, alongside the anti-inflammatory, antioxidant, and regulatory attributes of CURC on various oncogenic pathways, underscore the potential for a synergistic effect when these two agents are utilized together.

In cancer therapy, the ability of therapeutic agents to target specific sites, their specificity, potential adverse effects, and the capability of cells to resist treatment are crucial factors in disease management. The pharmacokinetic constraints of both compounds—swift onset of drug resistance with ATRA and low bioavailability, inadequate water solubility, and quick metabolic clearance of CURC—substantially diminish their clinical effectiveness [22,23,24,25]. Nanotechnology-based drug delivery systems can surmount these limitations, especially poor water-solubility, owing to their appropriate size, high stability, biodegradability, excellent biocompatibility, capacity for selective drug delivery, enhanced bioavailability with reduced side effects, low toxicity, and an ability to circumvent the liver, thereby avoiding first-pass metabolism [26,27,28]. Owing to their capacity to cross the BBB, these nanoscale systems have gained prominence in the advancement of GBM therapeutic research [3,29]. Nanotechnology-based drug delivery systems provide substantial benefits by enhancing the capacity of the drug to traverse the BBB and facilitating its regulated release, resulting in a more pronounced suppressive effect on GBM cells [30]. Progress in nano drug delivery systems has yielded a range of polymeric, lipid-based, or protein-based nanomaterials for glioma therapy [2].

Protein-based nanoparticles (NPs) are a compelling alternative to synthetic polymers owing to their biocompatibility, biodegradability, and minimal toxicity [31]. Among these natural carrier systems, albumin is highly favored for the targeted delivery of chemotherapy drugs, owing to its remarkable stability, substantial drug-binding capacity, chemically changeable structure, and the ability to transport hydrophobic compounds [31]. The capacity to aggregate around tumors through transcytosis via specific receptors offers a considerable tumor targeting advantage for albumin-based nanoformulations [32]. Bovine serum albumin (BSA) is garnering interest as a possible nanocarrier platform for improving treatment efficiency against problematic malignancies such as GBM, owing to its excellent compatibility with hydrophobic drugs and its ability to traverse the BBB [33]. With these advantages, BSA-based NPs serve as an ideal platform for the targeted delivery of ATRA and CURC.

In this work, we formulated biocompatible BSA-NPs co-loaded with ATRA and CURC to facilitate the targeted, efficient, and non-invasive delivery of these therapeutic compounds toward investigating their anti-GBM potential. While albumin-based nanoparticles have demonstrated clinical success in cancer therapy—most notably with nab-paclitaxel (Abraxane^®^) [34,35]—and curcumin-loaded nanocarriers have shown promise in preclinical glioblastoma models [36,37], the present study introduces a novel therapeutic approach that addresses critical gaps in existing strategies. To our knowledge, this is the first report of co-loaded ATRA–curcumin BSA nanoparticles specifically designed for glioblastoma therapy together with a notable lack in ATRA-based nanoformulations. This study advances beyond current albumin-based glioblastoma nanocarriers by combining ATRA with curcumin in a single platform that ensures synchronized delivery and optimal drug ratios.

## 2. Materials and Methods

### 2.1. Materials

Bovine Serum Albumin (A8806) and Glutaraldehyde (8.20603.1000) were purchased from Sigma-Aldrich (Schnelldorf, Germany) and Merck (Darmstadt, Germany), respectively. Absolute ethanol and all other solvents used were of analytical grade.

Human GBM cell line U87-GBM (ATCC HTB-14) was purchased from the American Type Culture Collection (ATCC), Manassas, VA, USA. All-trans retinoic acid (554720; Merck) and CURC (239802; Merck) were stored in accordance with the manufacturer’s instructions. ATRA and CURC were dissolved in DMSO (D2650; Sigma-Aldrich) per the manufacturer’s instructions to obtain stock solutions of 31.06 and 13.1451 mM, respectively. Dulbecco’s Modified Eagle’s Medium (DMEM; High Glucose with normal L-Glutamine, 500 ML) was the basal medium. To prepare the complete medium, 5 mL of 10% fetal bovine serum (F7524; Sigma-Aldrich) and 0.5 mL of 1% penicillin–streptomycin (15140122; Thermo Fisher) were added. The flasks were incubated at 37 °C under a humidified atmosphere consisting of 5% CO_2_. After covering 70–80% of the plate surface, the cells were harvested with trypsin-EDTA (25200056; Thermo Fisher Scientific, Waltham, MA, USA). Trypan blue (GF00376; GlpBio Technology Inc., Montclair, CA, USA) staining and a Cell Titer (Bio-Rad Laboratories, Hercules, CA, USA) were used for vital cell identification and counting, respectively. The vital cells at 1 × 10^6^ were passaged into flasks containing the complete medium.

### 2.2. Preparation and Optimization of BSA-NPs

**Figure 1:**
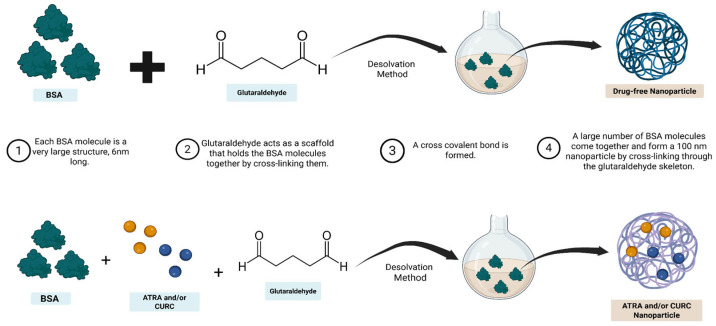
Synthesis of albumin-based NPs by the desolvation method and drug-loading (Created in BioRender. Sönmez, C. (2026) https://BioRender.com/mtbntjm). NP, nanoparticle.

**Figure 2:**
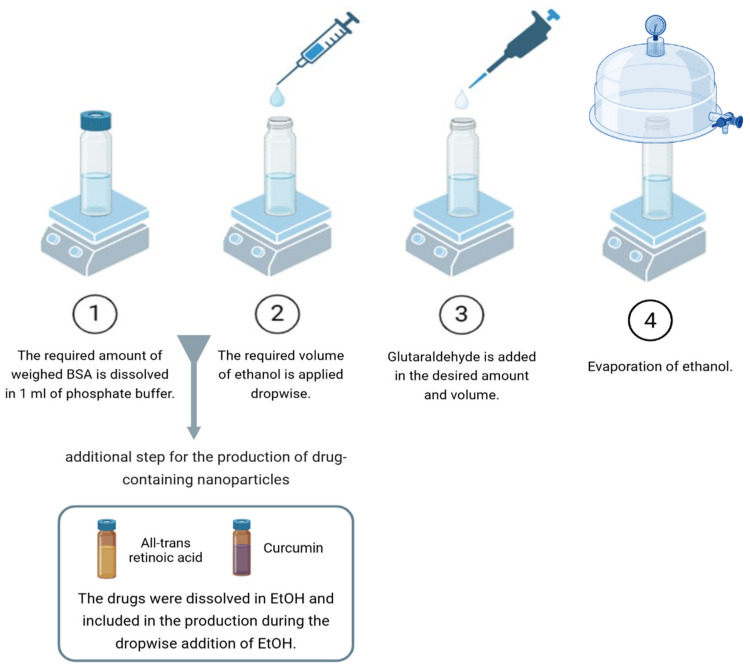
The stages of albumin NP production (Created in BioRender. Baltacıoğlu, A. (2026) https://BioRender.com/dg8wkbq). NP, nanoparticle.

The BSA-NPs were produced via the glutaraldehyde-based cross-linking of BSA molecules into a scaffold (Figure 1). ATRA and CURC-encapsulated BSA-NPs were synthesized using a well-established desolvation method [38,39], in which parameters such as the amounts of BSA and glutaraldehyde used, as well as the speed of solution mixing, are critical. Optimization is essential for this highly sensitive method to allow for drug binding and production in a usable form. Thus, the process of BSA-NP production was first optimized with nearly 23 formulations that did not contain the active ingredient (Tables S1 and S2). Optimization continued until NPs < 100 nm in size were obtained. Finally, the formulation with the smallest particles and lowest polydispersity index was selected as optimal. For this formulation, 30 mg of BSA was dissolved in 1 mL of phosphate buffer (pH 8.0), added with 0.75 mL of 50% ethanol (desolvation agent), 5% glutaraldehyde, and stirred at 500 rpm for 24 h. The production method is schematized in Figure 1. For synthesizing the drug-loaded NPs, 75 µM of ATRA and 20 µM of CURC were used together as well as separately to serve as controls. These doses were selected based on a preliminary study in which a synergistic interaction between the two drugs was observed (combination index, CI = 0.09409). Drugs were solubilized in 50% EtOH (desolvation agent) and incorporated into the formulation via dropwise addition (Figure 2).

### 2.3. Characterization of the ATRA-CURC-BSA–NPs

#### 2.3.1. Measurement of Particle Size (PS), Polydispersity Index, and Zeta Potential of the NPs

Particle size (PS), size distribution (polydispersity index, PDI), and zeta potential of the NPs were analyzed using the dynamic light scattering (DLS) method on a Dynapro Nanostar instrument (Wyatt Technology Corporation, Goleta, CA, USA), at 25 °C with a 90° side scatter angle. All samples were prediluted to different ratios with distilled water. During all measurements, the baseline and PDI values were set to 1.00 ± 0.01 and <0.3, respectively, and other parameters were considered unreliable. Each parameter was measured in at least six repetitions, and standard deviations (SDs) were obtained.

Zeta potential was analyzed with the same device, but by employing the Laser Doppler Velocimetry technique using an Omega cuvette at 25 °C.

#### 2.3.2. Evaluation of the Surface Morphology of NPs

The morphological properties of the ATRA-CURC-BSA–NPs were visualized by transmission electron microscopy (TEM; Talos L120C, Thermo-Fisher Scientific). The aqueous NP dispersion was diluted 100 times and then placed on a carbon-coated copper grid. The samples were completely air-dried at 25 °C. Images were captured under high-vacuum and low-pressure conditions [40].

#### 2.3.3. Purification of the ATRA-CURC-BSA–NPs and Determination of Encapsulation Efficiency (EE)

The EE of the ATRA-CURC-BSA–NPs was determined via an indirect method using the supernatants of the solutions obtained during each step of the NP preparation process. An Amicon filter with a molecular weight (MW) cut-off of 30 kDa and 15 mL volume was utilized for NP purification (UFC903008; Merck). Subsequently, the samples were passed through a 0.45 mm filter. A liquid chromatography–tandem mass spectrometry (LC-MS/MS) instrument was employed to quantify the drug load of the NPs. A TSQ Quantum Access Max system (Thermo-Fisher Scientific) with a C18 Hypersil Gold column (50 × 2.1 mm, 3 µm) was used. Water + 0.1% formic acid and acetonitrile + 0.1% formic acid served as the mobile phases, over a 6 min gradient at a flow rate of 0.7 mL/min. MS detection was operated in the positive ESI mode. The monitored MRM transitions were 301.4→122.9 (quantifier) and 301.3→283.1 (qualifier) for ATRA, and 369.4→177.1 (quantifier) and 369.0→285.0 (qualifier) for CURC.

#### 2.3.4. Determination of the Drug Release Profile of ATRA-CURC-BSA–NPs

The release of the drugs from the NP + drug combination group was examined using 20% EtOH at pHs 7.4 and 5.5. The NP suspension was introduced into a dialysis membrane with a MW cut-off of 3.5 kDa. The systems obtained were placed in a shaking incubator at 37 °C. The release media were sampled at 0, 15, 30, 45, and 60 min, in addition to days 1–15 [40]. They were quantitatively evaluated utilizing an LC-MS/MS device, as it specifically scans the total MW of the drug molecules and the MWs of the fragments unique to them, facilitated by the MS/MS detector.

### 2.4. Functional Experiments

#### 2.4.1. Cell Viability Assay

An xCELLigence Real-Time Cell Analysis (RTCA) Dual Purpose (DP) device was used. For background measurements, 50 µL of the medium was added to the E-plate. Then, the U87MG cells were prepared in 100 µL of medium and seeded into the wells (density: 5000 cells/well). The E-plates were incubated at room temperature for 30 min, and then placed in the device, which, in turn, was kept in an incubator, maintained under the standard conditions of 37 °C and 5% CO_2_ to ensure appropriate cell growth. After incubation for 24 h to allow attachment to the wells and entry into the logarithmic growth phase, the cells were treated with the NPs. The E-Plates were then reinserted into the device to monitor real-time cell growth. After 72 h of drug administration, the experiment was terminated, and the data were analyzed with the RTCAsoftware version 2.1.0 (Agilent Technologies, Santa Clara, CA, USA). All cell culture experiments were designed in triplicate.

#### 2.4.2. Migration Assay

The influence of ATRA-CURC-BSA–NPs on the migration of U-87 GBM cells was monitored in real time using the xCELLigence RTCA DP system with a CIM-Plate 16 (Agilent Technologies), following the manufacturer’s instructions.

U-87 cells were serum-starved in a medium containing 1% FBS, 12 h before the experiment. For the assay, 30,000 cells suspended in medium supplemented with 1% FBS were seeded into each well of the upper chamber (UC). The lower chamber (LC) was filled with a medium containing 10% FBS as a chemoattractant. The plate was incubated for 30 min. Then, the NPs were added to the designated wells (n = 3 per condition). Impedance values were recorded for >72 h, and the resulting cell index (CI) values were analyzed using GraphPad Prism software version 9.0 (https://www.graphpad.com/).

#### 2.4.3. Apoptosis Assay Using Annexin V/Propidium Iodide (PI) Flow Cytometry

The apoptotic effects of the NPs on U-87 MG cells were evaluated using an FITC Annexin V Apoptosis Detection Kit I (Cat. No: 556547; BD Biosciences, Franklin Lakes, NJ, USA) on a BD FACS Canto II flow cytometer (BD Biosciences). U-87 MG cells were seeded in 6-well plates at a density of 1 × 10^5^ cells per well. Next, the cells were incubated for 24 h, followed by treatment with each compound for 24, 48, and 72 h. For each time point, three biological replicates (n = 3) were prepared, and the cells were harvested for the analysis of apoptosis. First, the culture medium in each well was removed, and cells were washed with PBS. The medium and wash solution were transferred to collection tubes. Adherent cells were detached and then added to the respective tubes. These cell suspensions were centrifuged at 400× *g* for 5 min, and the supernatants were discarded. The cell pellets were washed with cold PBS and resuspended in 1X binding buffer per the manufacturer’s instructions. For staining, each sample was mixed with Annexin V-FITC and PI solutions provided in the kit at concentrations recommended by the manufacturer. The mixtures were gently vortexed and incubated in the dark at 25 °C for 15 min. The samples were then analyzed using a BD FACS Canto II flow cytometer. The data were processed and analyzed utilizing the FlowJo software version 10.10 (BD Biosciences).

### 2.5. Statistical Analysis

Cytotoxicity data are expressed as the mean ± standard deviation (SD). They were analyzed via two-way ANOVA followed by Tukey’s multiple comparisons test using GraphPad Prism version 9.0 (GraphPad Software, La Jolla, CA, USA). Migration data are presented as the median values with ranges. To evaluate the temporal inter-treatment group variations, a two-way ANOVA with Sidak’s multiple comparisons test was employed. A threshold of *p* < 0.05 was considered statistically significant.

## 3. Results

### 3.1. Preparation and Optimization of ATRA-CURC-BSA–NPs

**Figure 3:**
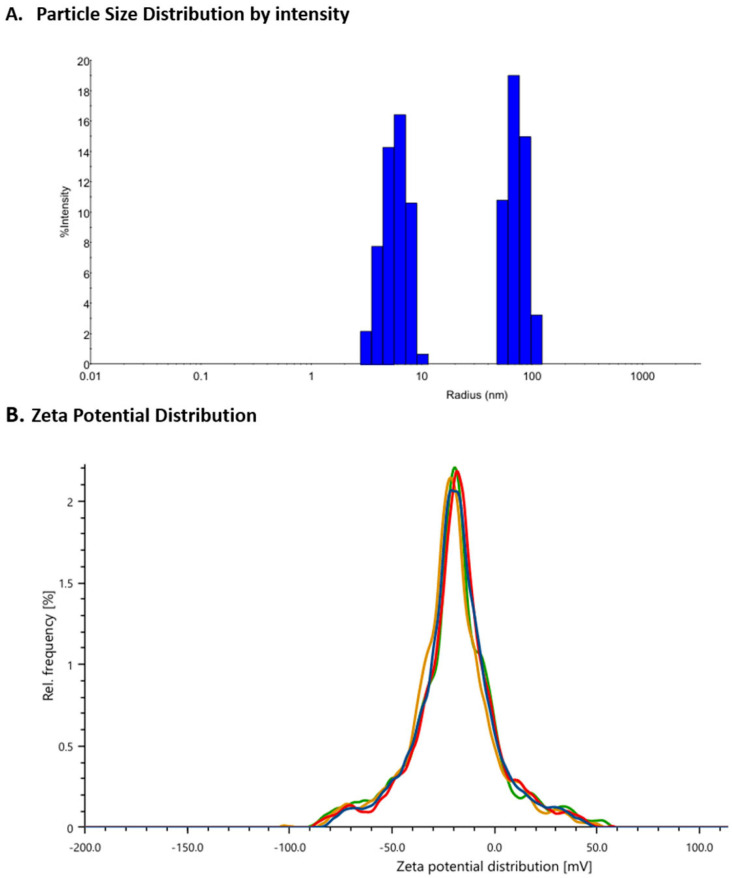
The PS (**A**) and zeta potential (**B**) distribution of the ATRA-CURC-BSA–NPs. BSA, bovine serum albumin; ATRA, all-trans retinoic acid; NP, nanoparticle; CURC, curcumin; PS, particle size.

This study aimed to obtain an ATRA-CURC-BSA–NP formulation exhibiting optimal EE, minimal PS, and a low PDI value. Following an assessment of the essential formulation and process parameters influencing these parameters during initial investigations, the ATRA-CURC-BSA–NPs were produced. The formulation optimization involved a rigorous evaluation of the BSA quantity, glutaraldehyde concentration, ethanol volume, stirring speed, and reagent addition rates. Each test formulation was characterized individually (Table S1). The preliminary experiments with elevated BSA (60 mg) and glutaraldehyde concentrations resulted in considerable aggregation and phase separation. Consequently, BSA was decreased to 30 mg, and the ethanol volume was optimized, with 0.75 mL identified as ideal. Particle production and stability were the best at a mixing speed of 500 rpm. The use of 2.5 mg glutaraldehyde dissolved in a total volume of 100 µL produced optimal results. A comparison of the reactant addition rates revealed that the dropwise addition of ethanol preserved the solution’s clarity. Conversely, the stage-wise addition of glutaraldehyde adversely impacted particle formation, and thus, a single application was deemed preferable. In the concluding phase, formulations containing several concentrations of ethanol were evaluated, and the one with 50% ethanol (C20) produced particles with superior characteristics (Table 1). Consequently, the C20 formulation was considered optimal, and the NPs produced using it were employed for the characterization studies (Figure S3). The PS and zeta potential distribution of the C20 ATRA-CURC-BSA–NPs are illustrated in Figure 3.

### 3.2. Determination of Encapsulation Efficiency (EE) and Drug Loading Capacity (DL)

BSA-NPs serve as robust and stable drug delivery devices owing to the covalent bonding of the protein. They possess significant stability and substantial drug loading capability [41,42]. In line with these findings, in this study, ATRA-CURC-BSA–NPs achieved a drug encapsulation effectiveness of 99% for the targeted dosages (Table 1). The ATRA and CURC concentrations were 45 and 15 µg/mL, respectively, indicating an elevated amount of drugs held by the ATRA-CURC-BSA–NPs. In addition to encapsulation efficiency, the drug loading capacity (DL) was calculated using the formula (drug/(BSA amount + drug)) × 100. Based on these calculations, the DL values were determined to be 0.15% for ATRA and 0.05% for curcumin.

### 3.3. Measurement of PS, PDI, and Zeta Potential

The C20 formulation was utilized for the fabrication of the drug-encapsulated NPs. Drug doses were established for ATRA at 75 µM, CURC at 20 µM, and a combination of the two. The PSs, PDI values, and zeta potentials of the NPs produced are summarized in Table S2. These characteristics indicated an increase in PS for the ATRA-CURC-BSA–NPs.

### 3.4. In Vitro Drug Release Studies

**Figure 4:**
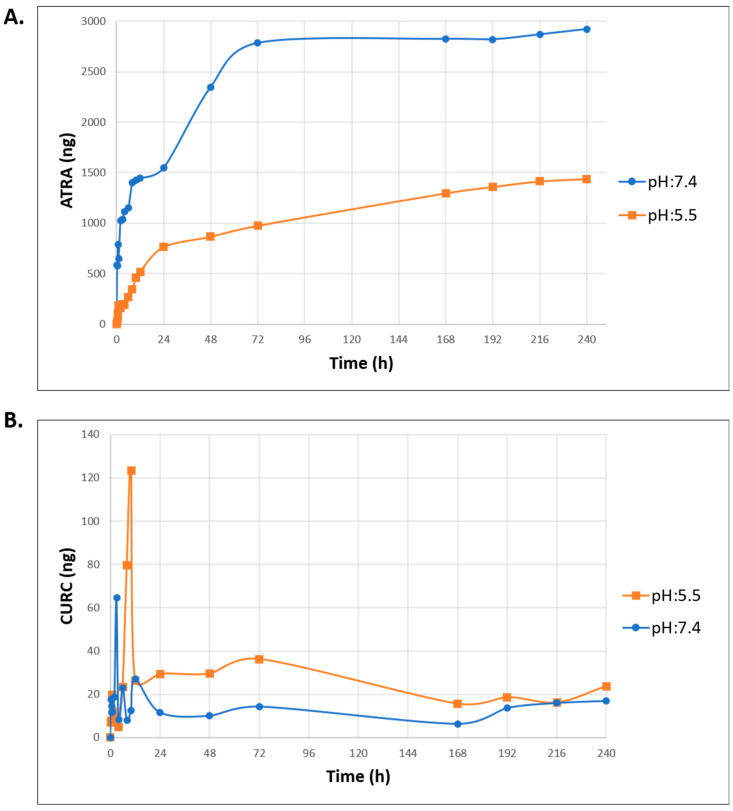
Drug release profile of ATRA-CURC-BSA–NPs in PBS–ethanol (80:20). Orange and blue lines represent pH 5.5 and 7.4, respectively. The quantities of ATRA (**A**) and CURC (**B**) released are shown in ng. BSA, bovine serum albumin; ATRA, all-trans retinoic acid; NP, nanoparticle; CURC, curcumin; PBS, phosphate-buffered saline.

The pH-dependent release behavior of ATRA and CURC from the ATRA-CURC-BSA–NPs was comprehensively monitored for 240 h under physiological (pH 7.4) and tumor-like acidic (pH 5.5) conditions, employing two dissolution media—PBS and PBS–ethanol (80:20) mixture, respectively. The low solubility of drugs in water has constrained their release in PBS medium (Figure S1). Figure 4 depicts the release profile of ATRA and CURC in a 20% ethanolic medium. The cumulative release was quantified and expressed in ng. ATRA exhibited a pronounced pH-sensitive release pattern, demonstrating a markedly greater cumulative release at pH 7.4 compared to pH 5.5 (Figure 4A). At pH 7.4, ATRA was initially released rapidly, reaching ~2800 ng by 72 h, with a total cumulative release of ~3000 ng at 240 h. Such a profile indicates that a bulk of the ATRA payload is discharged during the first 72 h, followed by a distinct plateau phase. Conversely, ATRA release at pH 5.5 followed a protracted and progressive profile, with the cumulative amount escalating steadily over the 240 h study duration, finally reaching ~1500 ng.

In contrast to the overall quantity dominance of ATRA, CURC release was primarily characterized by a remarkable initial burst effect at pH 5.5 (Figure 4B). At pH 5.5, a detailed analysis of the CURC release rate revealed a pronounced initial surge, which rapidly diminished within ~24 h, but showed a minor resurgence at ~72 h before stabilizing till the 240-h mark. At pH 7.4, the release rate was comparatively subdued. After a swift initial decrease, it stabilized, advancing at a gradual and consistent pace. Notably, despite the initially high CURC release burst at pH 5.5, the cumulative quantity of CURC released was substantially lower than that with ATRA across all tested conditions.

### 3.5. Evaluation of the Surface Morphology of NPs

**Figure 5:**
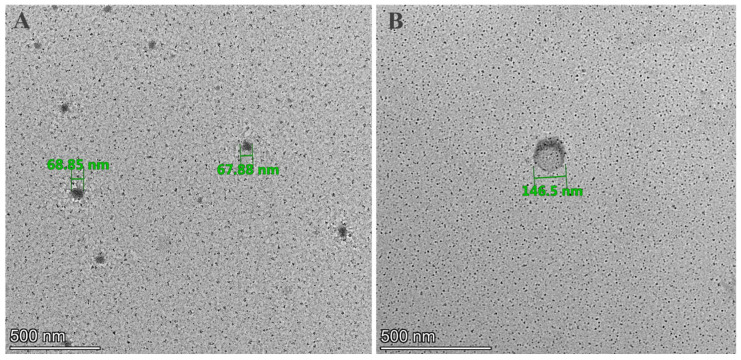
A TEM micrograph of the C20-based drug-free formulation (**A**) and ATRA-CURC-BSA–NPs (**B**). BSA, bovine serum albumin; ATRA, all-trans retinoic acid; NP, nanoparticle; CURC, curcumin; TEM, transmission electron microscopy; C20, optimized BSA nanoparticle formulation containing 50% ethanol.

The shape and morphological structure of the drug-free and drug-loaded BSA-NPs were investigated using TEM. As seen in the micrographs, the NPs displayed a uniformly spherical morphology with a monodispersed size distribution, without aggregation (Figure 5A), with the shape being preserved after drug loading (Figure 5B). Notably, the diameters of the drug-free and drug-loaded NPs (Figure 5A and Figure 5B, respectively) were slightly smaller in the TEM images than the corresponding hydrodynamic sizes obtained through DLS measurements. Such a well-documented discrepancy has been commonly reported by similar studies [43,44].

### 3.6. Cell Viability Assay

**Figure 6:**
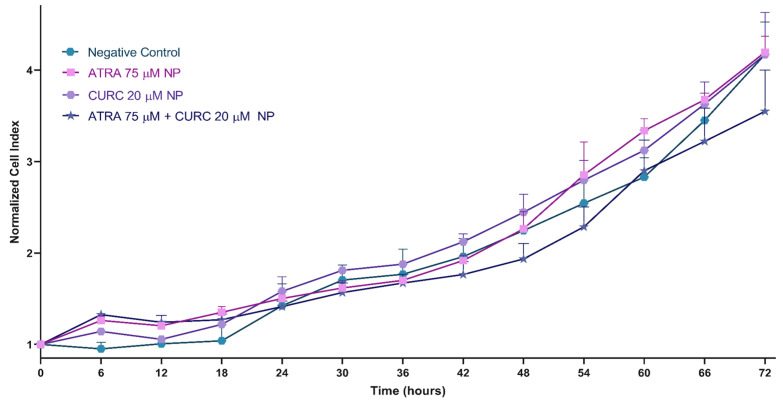
Impacts of drug-encapsulated BSA-NPs on the growth of U87-MG cells. The cells were treated with the corresponding BSA-NP formulations, with untreated cells serving as the control. CI was monitored for 72 h in real time using the xCELLigence DP system. Curves represent the means of the CI values obtained from three independent experiments (n = 3). BSA, bovine serum albumin; NP, nanoparticle; CI, cell index; DP, dual purpose (xCELLigence RTCA-DP system). Statistical comparisons versus the negative control were performed using two-way ANOVA with Sidak’s multiple comparisons; adjusted *p*-values are provided in Supplementary Table S3.

Real-time analyses demonstrated time-dependent alterations in the normalized CI during the 72 h study period across all groups. At the 24 and 48 h intervals, the proliferative activity of the ATRA-NP and CURC-NP groups was enhanced modestly. However, by 72 h, the proliferation levels converged with those of the negative control. These observations not only corroborate the sustained and regulated drug release behavior of the NP formulations but also parallel our findings mentioned in the previous sections, indicating that early exposure to ATRA or CURC can elicit a transient and mild proliferative response (Figure 6). However, the ATRA-CURC-BSA–NPs exhibited the lowest CI values starting from the 24th hour. Multiple comparisons based on two-way ANOVA with Sidak’s correction indicated significant differences at early time points for ATRA-NP (6 h: *p* = 0.005; 12 h: *p* = 0.002) and CURC-NP (6 h: *p* = 0.027) versus the negative control, whereas the co-loaded NP exhibited the most pronounced effect at 72 h (*p* < 0.001) (Supplementary Table S3).

### 3.7. Cell Migration Assay

**Figure 7:**
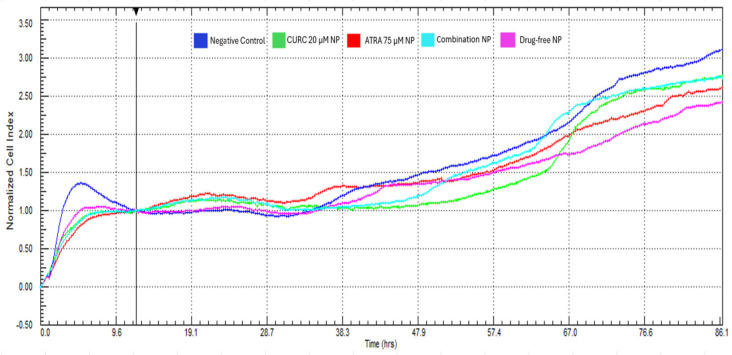
Real-time migration of U87-MG cells induced by the NP formulations. The migration was assessed using the xCELLigence Real-Time Cell Analysis Dual Purpose system for >86 h following a 12 h normalization period. The cells were treated with ATRA (75 µM)-NPs, curcumin (20 µM)-NPs, ATRA-CURC-BSA–NPs (combination), drug-free NPs (drug-free), and an untreated negative control. Subsequently assessed cell index values reflected the treatment effects. Data are presented as the means of three independent experiments (n = 3). Statistical comparisons versus the negative control and the drug-free NP were performed using two-way ANOVA with Sidak’s multiple comparisons; adjusted *p*-values are provided in Supplementary Table S4. BSA, bovine serum albumin; ATRA, all-trans retinoic acid; NP, nanoparticle; CURC, curcumin.

The real-time xCELLigence system was used to monitor the migration of the U87-MG cells for 86 h following a 12 h normalization period; the treatment effects were analyzed based on relative CI. In the negative control group, the CI increased steadily and reached ~3.4 by the end of the experiment, indicating uninterrupted migratory activity. The drug-free BSA-NPs produced a CI profile comparable to that of the control, suggesting no intrinsic impact on cell migration. In contrast, the ATRA-loaded NPs began to attenuate migration from ~30 h onward, with the CI values stabilizing at ~2.4. The CURC-loaded NPs exerted a similar inhibitory effect with a slightly later onset (~40 h), reaching a CI value of ~2.2. The NPs loaded with ATRA + CURC exhibited the most pronounced and sustained suppression, with CI values declining immediately post-normalization and stabilizing at ~2.0 at 86 h. Overall, these findings indicate that the NP formulations enhance the anti-migratory activity of ATRA and CURC, with the ATRA-CURC-BSA–NPs suppressing U87-MG cell migration most robustly (Figure 7).

Multiple comparisons (two-way ANOVA with Sidak’s correction) revealed time-dependent differences in U87-MG cell migration: CURC-NP showed the earliest significant reduction versus the negative control at 34–53 h (e.g., 34 h: *p* = 0.048; 37 h: *p* = 0.005; 49 h: *p* < 0.001), whereas ATRA-NP became significantly different from the negative control at later time points (e.g., 59 h: *p* = 0.024; 62 h: *p* < 0.001). In addition, drug-free NP differed from the negative control from 54 h onward (e.g., 54 h: *p* = 0.044; 57 h: *p* < 0.001), and the co-loaded NP exhibited significant differences versus the drug-free NP at 53–62 h (e.g., 53 h: *p* = 0.009; 54 h: *p* < 0.001; 60 h: *p* < 0.001) (Supplementary Table S4).

### 3.8. Apoptosis Assay

The capacity of the ATRA-CURC-BSA–NPs to induce apoptosis in U87 GBM cells was evaluated using Annexin V-FITC/PI double staining and flow cytometry. The experimental groups included a positive control (apoptosis-inducing factor), a negative control (no drug treatment), drug-free NPs, ATRA-loaded NPs, CURC-loaded NPs, and ATRA-CURC-BSA–NPs.

In the positive control group, 96.1% of cells were localized to the late apoptotic quadrant (Q3), confirming the effectiveness of the staining and gating strategy. In contrast, 96.7% of the cells in the negative control group remained viable (Q4), indicating minimal baseline programmed cell death (PCD) and validating the reliability of the analytical conditions (Supplementary Figure S2).

**Figure 8:**
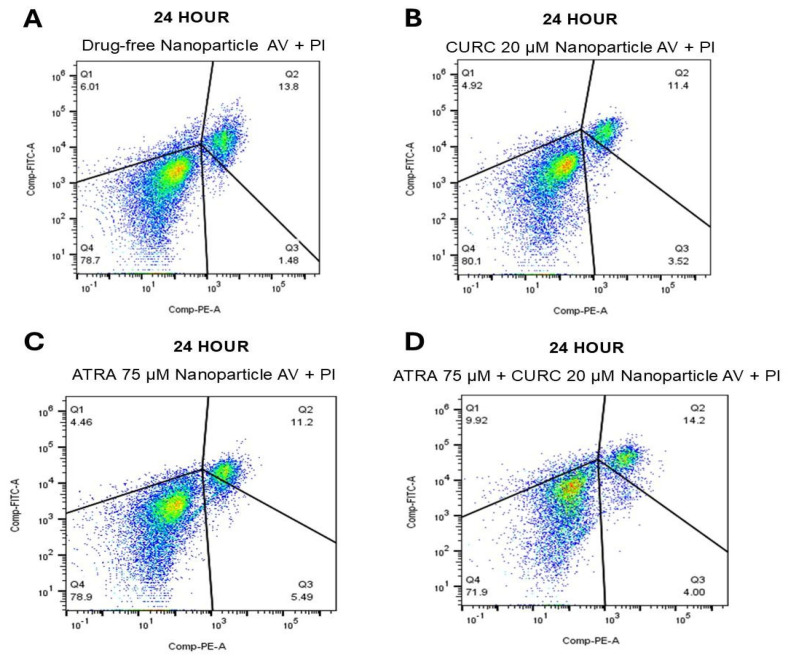
Apoptotic response of U87 GBM cells to NP formulations after 24 h. After 24 h, the cells were stained with Annexin V-FITC/PI and then analyzed by flow cytometry. (**A**): Drug-free NPs; (**B**): CURC (20 µM)-NPs; (**C**): ATRA (75 µM)-NPs; (**D**): Combination (ATRA 75 µM + CURC 20 µM)-NPs. GBM, glioblastoma (multiforme); NP, nanoparticle; CURC, curcumin; ATRA, all-trans retinoic acid.

At 24 h, flow cytometry revealed that a majority of the cells in all NP-treated groups remained viable at ~72–80% in Q4 (Figure 8). In the drug-free NP group, 78.7% of the cells were viable, with a small apoptotic fraction of ~ 7.5% (Q1: 6.0% and Q3: 1.5%). CURC-NP treatment yielded the highest cell viability (80.1%), accompanied by 8.4% apoptotic cells (Q1: 4.9% and Q3: 3.5%). In the ATRA-NP group, 78.9% of the cells were viable, and total apoptosis reached 9.9% (Q1: 4.5% and Q3: 5.5%). In the ATRA-CURC-BSA–NP group, cell viability declined to 71.9%, while the proportion of the apoptotic population increased to 13.9% (Q1: 9.9% and Q3: 4.0%), indicating that the combination condition induced the earliest detectable shift toward programmed cell death in the cells. At this early time point, the overall apoptosis remained limited, consistent with incomplete drug release from the NP formulations and a prominent contribution of non-specific/mechanical stress.

**Figure 9:**
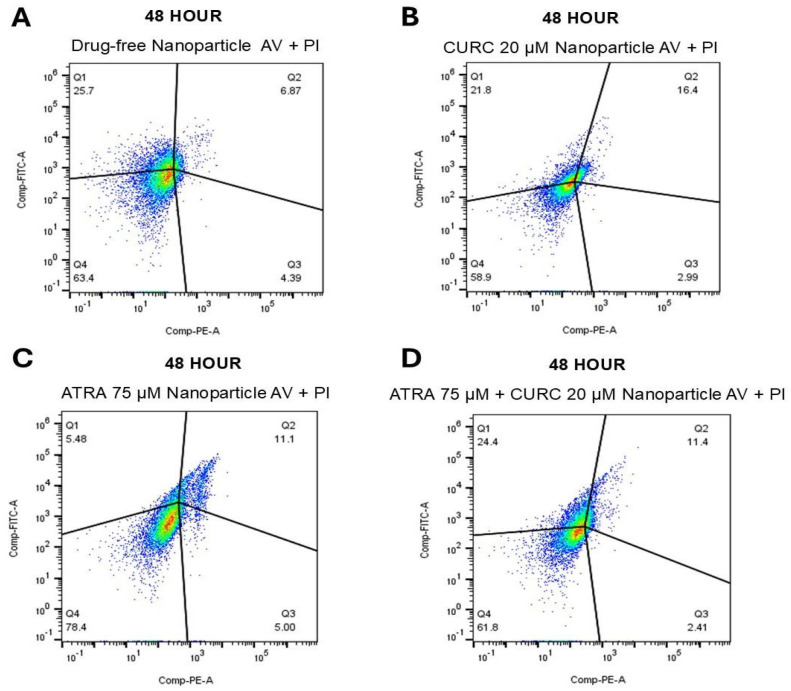
Apoptotic response of U87 GBM cells to NP formulations at 48 h. Cells were stained with Annexin V-FITC and PI at 48 h of drug treatment and evaluated by flow cytometry. (**A**): Drug-free NPs; (**B**): CURC (20 µM)-NPs; (**C**): ATRA (75 µM)-NPs; (**D**): Combination (ATRA 75 µM + CURC 20 µM)-NPs. GBM, glioblastoma (multiforme); NP, nanoparticle; CURC, curcumin; ATRA, all-trans retinoic acid.

The cytotoxic activity increased generally in all NP-treated groups at 48 h (Figure 9). In the drug-free NP group, cell viability declined to 63.4% (Q4), while total apoptosis rose to 30.1% (Q1: 25.7% and Q3: 4.39%) and necrosis reached 6.87% (Q2). CURC-loaded NPs reduced viability to a much greater extent of 58.9%, with 24.8% apoptotic cells (Q1: 21.8% and Q3: 2.99%) and 16.4% necrotic cells (Q2). In contrast, the ATRA-NP group preserved the maximal viability (78.4% in Q4), with a comparatively modest apoptotic fraction of 10.5% (Q1: 5.48% and Q3: 5.00%) and 11.1% necrosis (Q2). The ATRA-CURC-BSA–NP group exhibited viable cells at 61.8% and a 26.8% total apoptosis (Q1: 24.4% and Q3: 2.41%), with 11.4% necrotic cells (Q2), indicating a more pronounced shift toward apoptosis, particularly with the CURC or drug combination NPs.

**Figure 10:**
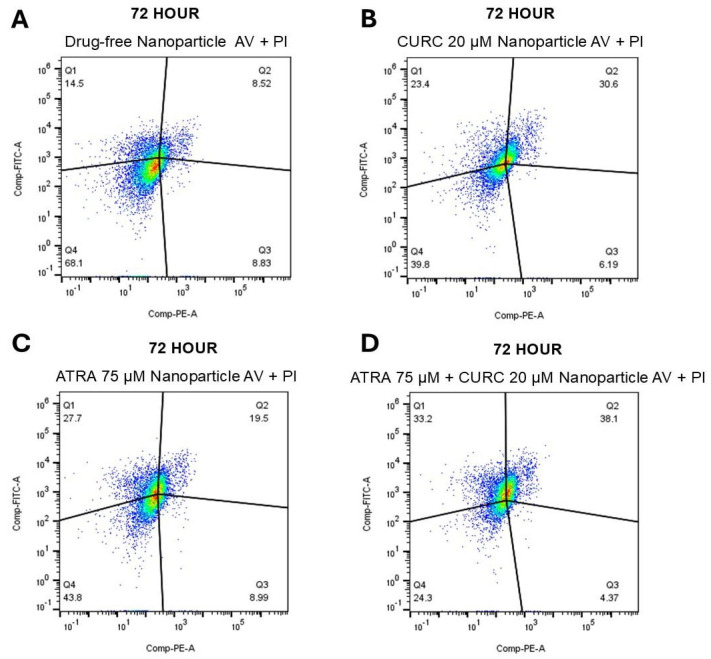
Apoptotic response of U87 GBM cells to NP formulations at 72 h. The cells were stained with Annexin V-FITC and PI at 72 h of drug administration and evaluated by flow cytometry. (**A**): Drug-free NPs; (**B**): CURC (20 µM)-NPs; (**C**): ATRA (75 µM)-NPs; (**D**): Combination (ATRA 75 µM + CURC 20 µM)-NPs. GBM, glioblastoma (multiforme); NP, nanoparticle; CURC, curcumin; ATRA, all-trans retinoic acid.

At 72 h, a clear time-dependent increase in apoptosis was identified across all NP-treated groups (Figure 10). In the drug-free NP group, 68.1% of the cells remained viable (Q4), whereas the total apoptotic fraction reached 23.3% (Q1: 14.5% and Q3: 8.83%). CURC NPs reduced viability to 39.8%, accompanied by apoptotic cells at 29.6% (Q1: 23.4% and Q3: 6.19%). The ATRA-NP group showed a total of 43.8% viable and 36.7% apoptotic cells (Q1: 27.7% and Q3: 8.99%). The ATRA-CURC-BSA–NPs demonstrated the most pronounced effects, reducing cell viability to 24.3% and yielding the highest apoptotic ratio of 37.6% (Q1: 33.2% and Q3: 4.37%). Collectively, these findings demonstrate that ATRA and CURC NP formulations induce a temporal apoptotic response in U87 GBM cells, with the drug combination NPs exerting the strongest pro-apoptotic influence compared with the single-drug NPs.

## 4. Discussion

Conventional cancer therapies—namely surgery, radiation, and systemic chemotherapy—frequently pose several obstacles that impede their efficacy. Lack of specificity because of miss-targeting cancer cells is a primary concern that restricts the effective dose of chemotherapeutic drugs [45,46,47]. Furthermore, the rise in drug resistance poses a critical challenge, as cancer cells evolve mechanisms to bypass the toxic effects of drugs [48]. Nanoparticle-based drug delivery systems have emerged as a revolutionary approach to overcome these biological barriers, offering enhanced efficacy and specificity of cancer therapies. NPs are designed to minimize off-target effects while enhancing the solubility of the bioactive ingredients and improving treatment strategies [49]. For instance, polymeric nanoparticles (PLGA, PEG, chitosan) offer tunable physicochemical properties, controlled release kinetics, and high drug-loading capacity highlighting characteristics of polymeric systems that can optimize tumor targeting [50,51,52,53]. However, polymeric nanoparticles face challenges related to scale-up complexity, batch-to-batch variability, potential immunogenicity, and regulatory hurdles associated with novel synthetic polymers [37,54]. Moreover lipid nanoparticles and liposomes enable encapsulation of both hydrophilic and hydrophobic compounds particularly critical for polyphenols like curcumin, which despite showing significant anti-cancer and anti-angiogenic properties in clinical trials, suffer from poor systemic bioavailability and rapid elimination [55,56,57]. Liposomal formulations of doxorubicin and irinotecan have been investigated in glioblastoma clinical trials, though results have been mixed due to limited BBB penetration without active targeting modifications [58,59]. Although liposomes offer easy functionalization with targeting ligands and have achieved clinical approval for multiple cancer indications, they suffer from limited tumor-specific targeting without surface modification, potential toxicity concerns, low drug loading capacity, and challenge of maintaining long-term stability [56,57,60]. Also, inorganic nanoparticles (gold nanoparticles, magnetic nanoparticles, mesoporous silica) provide unique properties including imaging capabilities, photothermal/photodynamic therapy potential, and high surface area for drug loading [61,62]. On the other hand, protein-based nanoparticles (albumin, ferritin) occupy a unique position in this landscape. Albumin nanoparticles offer enhanced receptor-mediated uptake through albumin-binding proteins, biocompatibility, natural biodegradability without toxic degradation products, intrinsic tumor-targeting properties, ease of modification and most importantly, established clinical precedent with nab-paclitaxel [63,64,65]. BSA-NPs are also garnering interest due to their ability to enhance the solubility and stability of hydrophobic drugs. This study focused on developing ATRA-CURC-BSA–NPs with high drug-loading efficiency and enhanced efficacy on U87 GBM cell lines. Compared to alternative platforms, BSA nanoparticles for ATRA–curcumin delivery offer clinical precedent supporting translational feasibility, natural biocompatibility and biodegradability, ability to co-load two hydrophobic agents with high efficiency, receptor-mediated tumor targeting without additional surface modification [66].

The study initially aimed to optimize the drug-free NP production method, addressing the key formulation parameters affecting their physicochemical characteristics. Excessive BSA and glutaraldehyde concentrations employed in the preliminary formulations led to particle aggregation, which was mitigated by lowering the BSA levels and adjusting the ethanol volume. The finalized C20 formulation produced particles with a size of 74.6 ± 9.3 nm, appropriate for GBM treatment, and exhibiting a zeta potential of −23 mV, indicating high colloidal stability (Table 1). Ethanol uses optimized reduced PS and enhanced uniformity [67]. As anticipated, the drug loading capacity was directly proportional to the PS, with the ATRA–CURC combination NPs reaching ~150 nm. This enlargement relative to the drug-free BSA-NPs may result from an interaction between the pharmaceuticals with the BSA matrix, influencing surface charge equilibrium and cross-linking density [68,69,70]. The translocation of NPs across the BBB is predominantly influenced by their physicochemical properties, particularly size and morphology. Particles smaller than ~200 nm interact more efficiently with the brain endothelial cells and display enhanced BBB penetration [46,47,48]. In our study, the ATRA-CURC-BSA–NPs had a mean diameter of ~150 nm and a uniformly spherical morphology (Figure 5), placing them within the 100–200 nm range reported to optimally facilitate superior cell uptake across the BBB [71], thereby supporting their suitability for targeting malignant GBM cells.

The release profile of the drugs can be modulated by their physicochemical characteristics, the NP formulation, and environmental factors such as pH and temperature [72]. BSA-NPs have been extensively studied for their potential applicability in controlled drug delivery systems due to their high stability, which facilitates a sustained and regulated release of the therapeutic agents. In particular, CURC, or paclitaxel-loaded BSA-based nanocarriers, substantially regulated the drug release kinetics, evidenced by the highly controlled, slow-release profiles—a cumulative release of only 36.76% and 27.2% at 48 h, respectively—underscoring the robust potential of BSA-NPs for the sustained delivery of various therapeutics [73,74]. Such an extended release might involve many mechanisms, including the hydrophilic and hydrophobic interactions promoted by BSA, which stabilize the NPs and impede drug transport [75]. Our study revealed a sustained release profile for the ATRA-CURC-BSA–NPs; however, the release medium induced a notable disparity in their release kinetics (Figure 4). Overall, ATRA release was higher, reaching ~3000 ng (70%), in contrast to that of CURC (Figure 4A). Specifically, the release rate of CURC reduced rapidly following an initial burst phase, which might be attributed to drug instability and degradation (Figure 4B). Additionally, the final cumulative release was greater at pH 7.4 with ATRA and pH 5.5 with CURC. Certain surface modifications in BSA can lead to less drug release at a low pH, while elevating it under the typical physiological pH of 7.4 [37,54]. Such a sensitivity underscores the influence of acidic environments on the release of pharmaceutical compounds from nanocarriers, suggesting rational design modifications to be crucial [75]. Specifically, engineering BSA-NPs by enhancing the opposing charges or hydrophobic interactions, or through the novel concept of integrating pH-sensitive polymers, can significantly improve targeted drug delivery [56]. This approach ensures greater drug stability outside the TME, especially with ATRA, thereby minimizing drug loss during systemic circulation and facilitating enhanced targeted release at specific pathological sites [16,76].

The xCELLigence RTCA DP device is an advanced system that allows the non-labeled real-time imaging of cell viability and migration. Its DP design allows functional experiments such as cell proliferation and migration. The device tests cell functions via impedance measurements performed using microelectronic sensors. Cytotoxicity results indicated that the CI was markedly suppressed by ATRA-CURC-BSA–NPs compared to the negative control within the exposure duration of 72 h (Table S3). However, ATRA- or CURC-NPs did not show any significant inhibitory effects. These results validated a sustained and controlled drug release pattern. Moreover, CURC diminished the migration and invasion ability of GBM cells by suppressing the inflammation as well as proliferation-associated signaling pathways, including NF-κB and PI3K/Akt, while simultaneously enhancing the pro-apoptotic response [40]. In this study, CURC-loaded NPs markedly inhibited migration from the 40th hour onward (Figure 7). The findings also suggested that NP-based formulations enhanced the bioavailability of CURC, improving cell uptake and inducing a potent anti-cancer response. Similarly, CURC encapsulated in solid lipid NPs exerted a substantial anti-cancer effect by markedly enhancing its bioavailability and efficacy [14]. ATRA modulates the expression of matrix metalloproteinases (MMPs), diminishing cell migration and invasion while promoting cell differentiation and death. Our study revealed a considerably decelerated migration in the ATRA-loaded NP group, particularly after the 30th hour (Figure 7), which may be associated with its capability to inhibit MMP-2/9 production and a partial reversal of EMT [77,78].

The dimensions of the drug-free NPs were inferior in our formulations, compared to the suspension. The density and composition of NPs influence their interaction with cells. High-density NPs may engage more aggressively with the cell membranes, leading to mechanical stress-induced necrosis and inflammatory responses. Such an interpretation is supported by our Annexin V-FITC/PI results, which showed low levels of apoptosis and necrosis in the drug-free NP group during the first 24 h (Figure 8), followed by a marked shift toward a predominantly apoptotic, rather than necrotic, cell death profile in the drug-loaded groups between 48 (Figure 9) and 72 h (Figure 10). Although ATRA-NP and CURC-NP caused limited changes in real-time Cell Index (Figure 6), Annexin V/PI assays detected apoptotic signaling for the same treatments (Figure 8, Figure 9 and Figure 10). This is not necessarily contradictory because RTCA Cell Index reflects not only cell number but also adhesion/morphology, while Annexin V detects early apoptosis before loss of membrane integrity and overt detachment. The gradual increase in early and late apoptotic cell populations in the ATRA-CURC-BSA–NP group was consistent with the controlled onset of drug release from the BSA matrix and the predominant occurrence of PCD at later time points.

The effects of the ATRA-CURC-BSA–NP formulations in U87-MG GBM cells indicated that BSA-based carrier systems might enhance the bioavailability of both compounds, facilitating modified controlled and sustained release, thereby demonstrating a potential for long-term efficacy without early-stage toxicity. These findings indicate the ATRA-CURC-BSA system as a promising candidate for advanced preclinical investigations.

Collectively, these results support the potential of the ATRA-CURC-BSA system as a platform for future preclinical investigations. The developed carrier system possesses significant potential in improving the transport of anticancer drugs and mitigating the associated constraints. Continued research aimed at investigating their pharmacological effects and tissue distribution in animal models can enhance the translational potential of this promising drug delivery system in GBM therapy.

This study has limitations, particularly the lack of in vivo validation and unaddressed challenges related to blood–brain barrier (BBB) transport. In vitro findings cannot fully predict therapeutic efficacy in the complex glioblastoma microenvironment, and BBB penetration—the most critical obstacle for brain tumor therapy—was not experimentally validated. Although nanoparticles within the 50–200 nm size range have been reported to be optimal for crossing the blood–brain barrier, and albumin-based nanoparticles are capable of traversing the BBB via gp60 (albondin) receptor-mediated transcytosis followed by caveolin-dependent transport across the endothelial barrier, rigorous in vivo biodistribution studies are essential to confirm brain accumulation and to accurately assess their clinical translational potential [79,80,81].

## 5. Conclusions

This study successfully demonstrates the development and optimization of a novel bovine serum albumin (BSA) nanoparticle (NP) platform designed for the synchronized delivery of all-trans retinoic acid (ATRA) and curcumin (CURC) to combat glioblastoma (GBM). The optimized desolvation method yielded nanocarriers with an exceptional encapsulation efficiency of over 99% and a mean particle size of approximately 150 nm, placing them within the ideal range for potentially traversing the blood–brain barrier (BBB) and achieving effective cellular uptake. Functional assays on U87-MG cells revealed that the delivery of ATRA-CURC-BSA–NPs provides an enhanced antitumor response compared to single-drug-NP treatments, effectively suppressing cell proliferation and significantly inhibiting migratory activity. Furthermore, the system’s controlled release profile facilitated a temporal shift in programmed cell death, moving from early to late apoptosis. As the first study to investigate the co-delivery of ATRA and curcumin via an albumin-based nanoplatform for glioblastoma, this research confirms that protein-based carriers can stand out as a biocompatible and multi-targeted drug delivery platform that effectively enhances the therapeutic potential of the encapsulated agents. While the in vitro results are highly promising, future in vivo investigations are necessary to confirm the system’s ability to penetrate the BBB and validate its therapeutic efficacy within the complex tumor microenvironment.

## Figures and Tables

**Table 1:** DLS measurements and EE of the NPs.

Formulation	PS (nm)	PDI	Zeta Potential (mV)	EE (%)
C20 (Free drug)	74.69 ± 9.3	0.24 ± 0.0	−23.025 ± 0.8	-
ATRA-loaded NP	104.4 ± 9.9	0.29 ± 0.05	−17.3 ± 3.8	99.679
CURC-loaded NP	95.0 ± 8.6	0.31 ± 0.06	−23.6 ± 0.2	99.991
ATRA-CURC-BSA–NP	154.7 ± 9.8	0.31 ± 0.08	−18.2 ± 2.5	99.999 (ATRA)
				99.996 (CURC)

DLS, dynamic light scattering; PDI, polydispersity index; PS, particle size; EE, encapsulation efficiency; BSA, bovine serum albumin; C20, optimized BSA nanoparticle formulation containing 50% ethanol; ATRA, all-trans retinoic acid; NP, nanoparticle; CURC, curcumin.

## Data Availability

The raw data supporting the conclusions can be provided by the authors on request.

## Supplementary Materials

The following supporting information can be downloaded at: https://www.mdpi.com/article/10.3390/life16010131/s1. Figure S1: Release profile of combination loaded BSA-NPs in PBS medium both pH:5.5 (orange line) and in pH:7.4 (blue line). Figure S2: Annexin V-FITC/PI analysis of positive and negative control U87 GBM cells. Figure S3: The particle size and zeta potential distribution of the C20 (drug-free) formulation. Table S1: Formulations prepared during the optimization process. Table S2: Characterization of drug-free formulations prepared during the optimization process. Table S3: Cytotoxicity results of the multiple ANOVA (Sidak) comparison test for the nanoparticle groups. Table S4: Migration results of the multiple ANOVA (Sidak) comparison test for the nanoparticle groups.

## Abbreviations

The following abbreviations are used in this manuscript:

Akt (AKT)	Serine/threonine-specific protein kinase Akt (Protein kinase B)
ANOVA	Analysis of variance
ATCC	American Type Culture Collection
ATRA	All-trans retinoic acid
ATRA-NP	ATRA-loaded nanoparticle
BBB	Blood–brain barrier
BSA	Bovine serum albumin
BSA-NP	Bovine serum albumin–based nanoparticle
C18	Octadecyl (C18) reversed-phase chromatography column
C20	Optimized BSA nanoparticle formulation containing 50% ethanol
CI	Cell index
CIM-Plate 16	16-well cell invasion and migration plate used with xCELLigence
CO_2_	Carbon dioxide
CURC	Curcumin
CURC-NP	Curcumin-loaded nanoparticle
DLS	Dynamic light scattering
DMEM	Dulbecco’s Modified Eagle’s Medium
DMSO	Dimethyl sulfoxide
DP	Dual Purpose (xCELLigence RTCA-DP system)
EDTA	Ethylenediaminetetraacetic acid
EE	Encapsulation efficiency
EMT	Epithelial–mesenchymal transition
EtOH	Ethanol
FACS	Fluorescence-activated cell sorting
FBS	Fetal bovine serum
FITC	Fluorescein isothiocyanate
GBM	Glioblastoma (multiforme)
GSC	Glioblastoma stem cell
HPLC	High-performance liquid chromatography
ICH	International Council on Harmonization
LC	Lower chamber (migration assay)
LC-MS/MS	Liquid chromatography–tandem mass spectrometry
MCF-7	Human breast adenocarcinoma cell line MCF-7
MMP	Matrix metalloproteinase
MMP-2	Matrix metalloproteinase-2 (gelatinase A)
MMP-9	Matrix metalloproteinase-9 (gelatinase B)
NF-κB	Nuclear factor kappa-light-chain-enhancer of activated B cells
NP	Nanoparticle
PBS	Phosphate-buffered saline
PDI	Polydispersity index
PI	Propidium iodide
PI3K	Phosphoinositide 3-kinase
PS	Particle size
Q1	Flow cytometry quadrant 1 (Annexin V^+^/PI^+^; late apoptosis/secondary necrosis)
Q2	Flow cytometry quadrant 2 (PI^+^; necrotic/late apoptotic cells)
Q3	Flow cytometry quadrant 3 (Annexin V^+^/PI^−^; early apoptotic cells)
Q4	Flow cytometry quadrant 4 (Annexin V^−^/PI^−^; viable cells)
Rb	Retinoblastoma protein
rpm	Revolutions per minute
RTCA	Real-Time Cell Analyzer
RTK	Receptor tyrosine kinase
SD	Standard deviation
STAT3	Signal transducer and activator of transcription 3
TEM	Transmission electron microscopy
TME	Tumor microenvironment
TMZ	Temozolomide
U87-MG	Human glioblastoma cell line U87-MG (ATCC HTB-14)
UV	Ultraviolet
UC	Upper Chamber (migration assay)
WHO	World Health Organization
Wnt/β-catenin	Wnt/β-catenin signaling pathway
xCELLigence	Real-time cell analysis system (xCELLigence RTCA)
