# Supplementary material for: Antitumor Activity of All-Trans Retinoic Acid and Curcumin-Loaded BSA Nanoparticles Against U87 Glioblastoma Cells
Source: Life (Basel). 2026 Jan 15;16(1):131. doi: 10.3390/life16010131 (PMC12842954; doi:10.3390/life16010131)
Supplement: Supplementary file 1 [file life-16-00131-s001.zip › life-4049155-supplementary.pdf]

Supplementary Table S1. Formulations prepared during the optimization process (BSA, phosphate buffer, ethanol, glutaraldehyde concentration, stirring speed and reaction time).

	**BSA (mg)**	**Phosphate buffer (mL)**	**Ethanol (mL)**	**Glutaraldehyde**	**rpm**	**Reaction time**
**C1**	60	1	-	10 mg<br>20 µl	500	24 hour
**C2**	30	1	1	10 mg<br>20 µl	500	24 hour
**C3**	30	1	0.75	10 mg<br>20 µl	500	24 hour
**C4**	30	1	0.5	10 mg<br>20 µl	500	24 hour
**C5**	30	1	0.25	10 mg<br>20 µl	500	24 hour
**C6**	30	1	0.1	10 mg<br>20 µl	500	24 hour
**C7**	30	1	0.75	10 mg<br>20 µl	200	24 hour
**C8**	30	1	0.75	10 mg<br>20 µl	300	24 hour
**C9**	30	1	0.75	10 mg<br>20 µl	750	24 hour
**C10**	30	1	0.75	10 mg<br>20 µl	1000	24 hour

**C11**	30	1	0.75	2.5 mg<br>40 µl	500	24 hour
**C12**	30	1	0.75	5 mg<br>40 µl	500	24 hour
**C13**	30	1	0.75	10 mg<br>40 µl	500	24 hour
**C14**	30	1	0.75	15 mg<br>40 µl	500	24 hour
**C15**	30	1	0.75	20 mg<br>40 µl	500	24 hour
**C16**	30	1	0.75	2.5 mg<br>30 µl	500	24 hour
**C17**	30	1	0.75	2.5 mg<br>50 µl	500	24 hour
**C18**	30	1	0.75	2.5 mg<br>75 µl	500	24 hour
**C19**	30	1	0.75	2.5 mg<br>100 µl	500	24 hour
**C20**	30	1	0.75<br>%50 EtOH	2.5 mg<br>100 µl	500	24 hour
**C21**	30	1	0.75<br>%70 EtOH	2.5 mg<br>100 µl	500	24 hour

**C22**	30	1	0.75<br><br>%96<br>EtOH	2.5 mg<br><br>100 µl	500	24 hour
**C23**	30	1	0.75<br><br>%99<br>EtOH	2.5 mg<br><br>100 µl	500	24 hour

A.

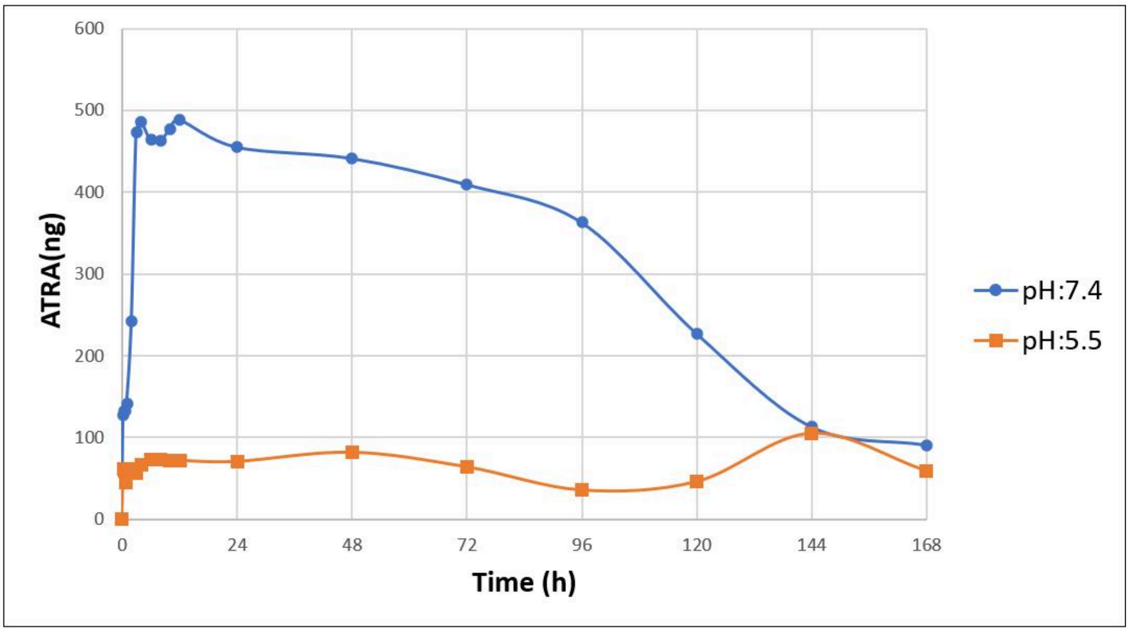

B.

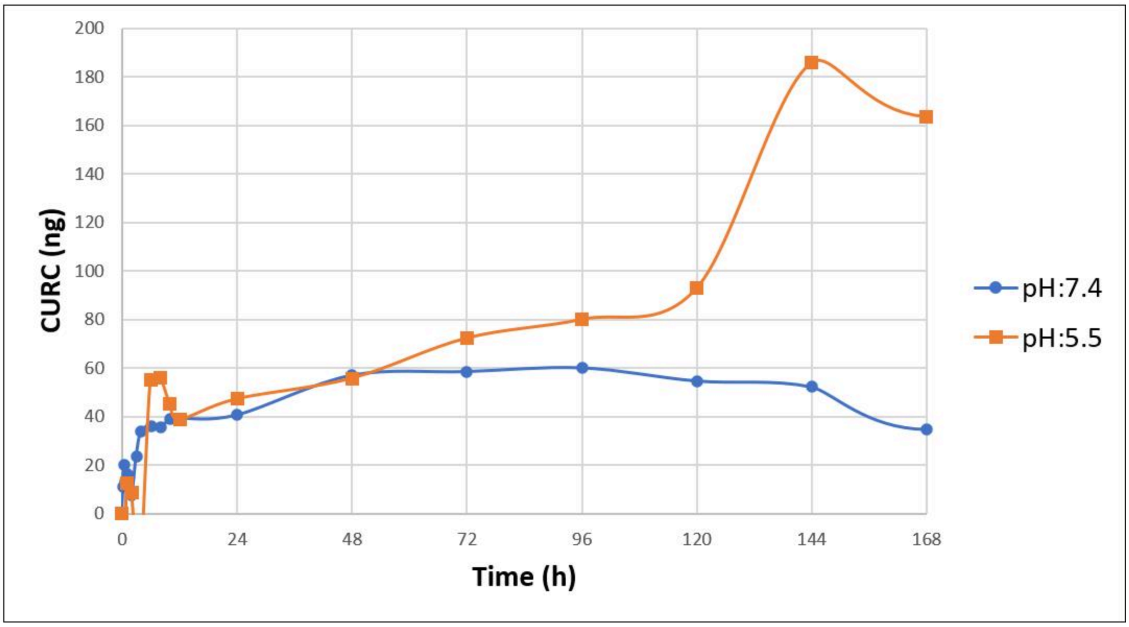

Supplementary Figure S1. Release profile of combination loaded BSA nanoparticles in PBS medium both pH:5.5 (orange line) and in pH:7.4 (blue line). The released ATRA (A) and curcumin (B) drugs are shown in nanograms.

Supplementary Table S2. Characterization of drug-free formulations prepared during the optimization process (Particle Size, PDI, zeta potential)

**Formulation Code**	**Particle size (nm) ± SD**	**Polydispersite Index (PDI) ± SD**	**Zeta Potential (mV) ± SD**
**C1**	2555.2±1841.9	0.3±0.0	-30.6±0.8
**C2**	649.3±165.9	0.3±0.0	-30.6±0.8
**C3**	485.1±70.4	0.3±0.0	-24.4±0.5
**C4**	572.1±145.1	0.3±0.1	-15.5±1.0
**C5**	616.4±76.0	0.3±0.0	-37.8±8.3
**C6**	457.7±50.9	0.3±0.0	-26.2±1.8
**C7**	422.4±45.1	0.3±0.0	-29.1±3.4
**C8**	585.7±112.8	0.4±0.2	-25.5±1.0
**C9**	520.8±114.0	0.3±0.0	-33.9±2.4
**C10**	814.0±409.4	0.3±0.0	-28.0±0.6
**C11**	154.8±24.1	0.2±0.0	-31.7±1.8
**C12**	224.5±5.8	0.2±0.0	-33.2±2.1
**C13**	261.3±6.8	0.2±0.0	-32.3±0.9
**C14**	399.5±87.7	0.3±0.0	-23.3±6.0
**C15**	495.8±98.6	0.3±0.0	-29.1±6.0
**C16**	161.0±16.3	0.3±0.2	-0.9±2.5

**C17**	94.7±25.1	0.3±0.0	-17.3±9.4
**C18**	94.2±12.5	0.3±0.0	-23.0±5.2
**C19**	93.8±4.4	0.2±0.0	-2.4±1.1
**C20**	74.69±9.,3	0.24±0.0	-23.02±0.8
**C21**	153.0±2.7	0.2±0.0	-26.0±1.3
**C22**	208.2±2.6	0.2±0.0	-25.5±1.2
**C23**	180.2±3.2	0.2±0.0	-24.2±3.6

SD, Standard Deviation.

Supplementary Table S3. Cytotoxicity results of the multiple ANOVA (Sidak) comparison test for the nanoparticle groups. Adjusted p-values obtained from Sidak's multiple comparisons test for U87-MG cell cytotoxicity show the effects of ATRA (75 μM) NP, curcumin (20 μM) fNP, and co-loaded nanoparticle groups compared with the negative control group at different time points (0–72 h). "ns" (non-significant) indicates non-significant differences, whereas $p < 0.05$, $p < 0.01$, and $p < 0.001$ indicate statistically significant differences.

Time (h)	ATRA 75 µM NP vs Negative Control	CURC 20 µM NP vs Negative Control	ATRA 75 µM + CURC 20 NP vs Negative Control
0 h	ns	ns	ns
6 h	0.005**	0.027*	0.004**
12 h	0.002**	ns	0.017*
18 h	ns	ns	ns
24 h	ns	ns	ns
30 h	ns	ns	ns
36 h	ns	ns	ns
42 h	ns	ns	ns
48 h	ns	ns	ns
54 h	ns	ns	ns
60 h	0.003**	ns	ns
66 h	ns	ns	ns
72 h	ns	ns	<0.001***

Supplementary Table S4. Migration results of the multiple ANOVA (Sidak) comparison test for the nanoparticle groups. Adjusted p-values obtained from Sidak's multiple comparisons test for U87-MG cell migration show the effects of ATRA (75 µM) NP, curcumin (20 µM) NP, and co-loaded nanoparticle groups compared with the negative control and the drug-free NP group at different time points (0–87 h). "ns" (non-significant) indicates non-significant differences, whereas $p < 0.05$, $p < 0.01$, and $p < 0.001$ indicate statistically significant differences.

Time (h)	ATRA 75 µM NP vs Negative Control	CURC 20 µM NP vs Negative Control	ATRA 75 µM + CURC 20 NP vs Negative Control	Drug-free NP vs Negative Control	ATRA 75 µM NP vs Drug-free NP	CURC 20 µM NP vs Drug-free NP	ATRA 75 µM + CURC 20 NP vs Drug-free NP
0 h - 33h	ns	ns	ns	ns	ns	ns	ns
34 h	ns	0.048*	ns	ns	ns	ns	ns
35 h	ns	0.023*	ns	ns	ns	ns	ns
36 h	ns	0.029*	ns	ns	ns	ns	ns
37 h	ns	0.005**	ns	ns	ns	ns	ns
38 h	ns	0.005**	ns	ns	ns	ns	ns
39 h	ns	0.003**	ns	ns	ns	ns	ns
40 h	ns	0.002**	ns	ns	ns	ns	ns
41 h	ns	0.001**	ns	ns	ns	ns	ns
42 h	ns	0.001**	ns	ns	ns	ns	ns
43 h	ns	0.001**	ns	ns	ns	ns	ns
44 h	ns	0.001**	ns	ns	ns	ns	ns

45 h	ns	0.001**	ns	ns	ns	ns	ns
46 h	ns	0.001**	ns	ns	ns	ns	ns
47 h	ns	0.001**	ns	ns	ns	ns	ns
48 h	ns	0.001**	ns	ns	ns	ns	ns
49 h	ns	<0.001***	ns	ns	ns	ns	ns
50 h	ns	<0.001***	ns	ns	ns	ns	ns
51 h	ns	<0.001***	ns	ns	ns	ns	ns
52 h	ns	<0.001***	ns	ns	ns	ns	ns
53 h	ns	<0.001***	ns	ns	ns	ns	0.009*
54 h	ns	0.032*	ns	0.044*	ns	ns	<0.001***
55 h	ns	ns	ns	0.017*	ns	ns	<0.001***
56 h	ns	ns	ns	0.001**	ns	ns	<0.001***
57 h	ns	ns	ns	<0.001***	0.036*	0.008**	<0.001***
58 h	ns	ns	ns	<0.001***	ns	0.002**	<0.001***
59 h	0.024*	ns	ns	<0.001***	ns	0.001**	<0.001***
60 h	0.017*	ns	ns	<0.001***	ns	<0.001***	<0.001***
61 h	0.001**	ns	ns	<0.001***	ns	<0.001***	<0.001***
62 h	<0.001***	ns	ns	<0.001***	ns	<0.001***	<0.001***
63 h	0.001**	ns	ns	<0.001***	ns	0.001**	0.001**

64 h	0.001**	ns	ns	<0.001***	ns	0.002**	0.001**
65 h	0.001**	ns	ns	<0.001***	ns	0.006**	0.002**
66 h	0.004**	ns	ns	<0.001***	ns	0.012*	0.003**
67 h	0.007**	ns	ns	<0.001***	ns	0.027*	0.006**
68 h	0.006**	ns	ns	<0.001***	ns	0.032*	0.029*
69 h	0.005**	ns	ns	<0.001***	ns	0.045*	0.040*
70 h	0.002**	ns	ns	<0.001***	ns	0.037*	0.049*
71 h	<0.001***	ns	ns	<0.001***	ns	0.034*	0.032*
72 h	<0.001***	0.038*	0.043*	<0.001***	ns	0.049*	0.043*
73 h	<0.001***	0.023*	0.014*	<0.001***	ns	ns	ns
74 h	<0.001***	0.008**	0.005**	<0.001***	ns	ns	ns
75 h	<0.001***	0.004**	0.002**	<0.001***	ns	ns	ns
76 h	<0.001***	0.001**	<,001***	<0.001***	ns	ns	ns
77 h	<0.001***	<0.001***	<0.001***	<0.001***	ns	ns	ns
78 h	<0.001***	<0.001***	<0.001***	<0.001***	ns	ns	ns
79 h	<0.001***	<0.001***	<0.001***	<0.001***	ns	ns	ns
80 h	<0.001***	<0.001***	<0.001***	<0.001***	ns	ns	ns
81 h	<0.001***	<0.001***	<0.001***	<0.001***	ns	ns	ns
82 h	<0.001***	<0.001***	<0.001***	<0.001***	ns	ns	ns

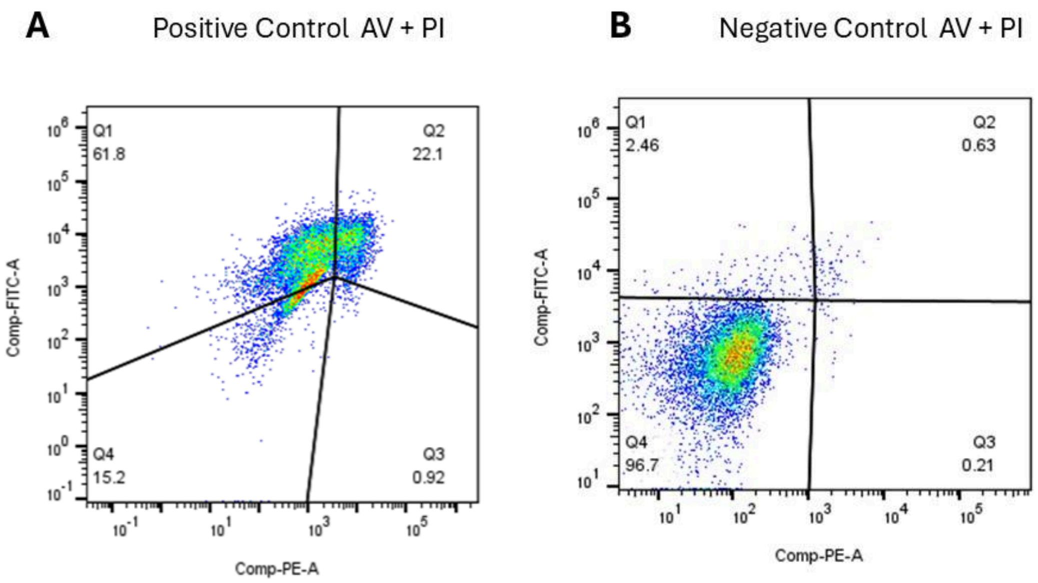

Supplementary Figure S2. Annexin V-FITC/PI analysis of positive and negative control U87 glioblastoma cells. U87 cells were either exposed to an apoptosis-inducing positive control condition or left untreated as a negative control and then stained with Annexin V-FITC and propidium iodide (PI). A: Positive control; B: Negative control.

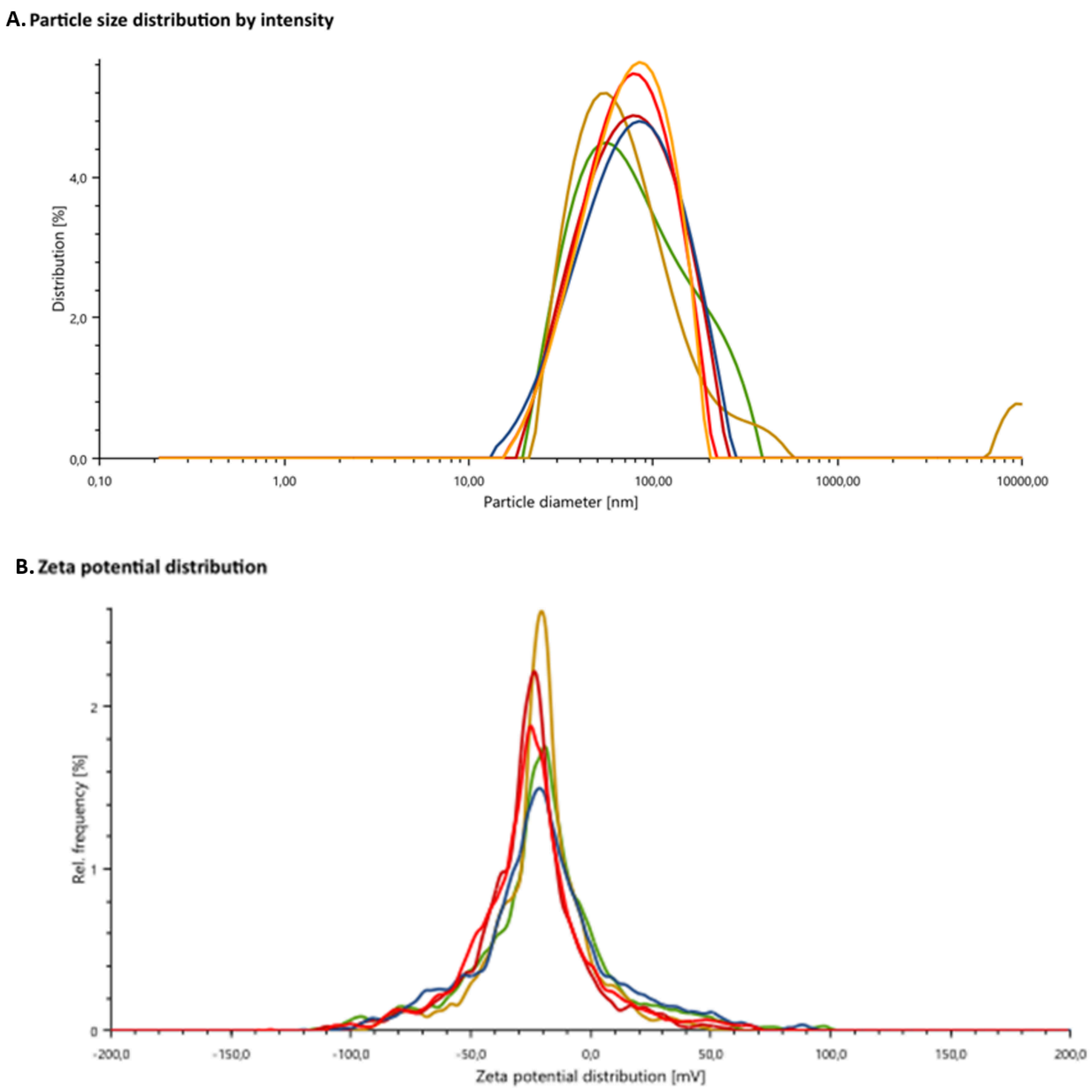

Supplementary Figure S3. The particle size (A) and zeta potential (B) distribution of the C20 (drug-free) formulation.
